# Seroma of Auricle

**DOI:** 10.7759/cureus.31200

**Published:** 2022-11-07

**Authors:** Hrishita Shirsath, Shraddha Jain

**Affiliations:** 1 Otolaryngology, Jawaharlal Nehru Medical College, Datta Meghe Institute of Medical Sciences, Wardha, IND

**Keywords:** pinna swelling, ear cyst, intralesional steroid injection, aspiration, surgical deroofing, auricular seroma, auricular pseudocyst

## Abstract

The pseudocyst of the auricle is an intracartilaginous lesion defined as the accumulation of sterile, straw-coloured fluid in a cyst unlined by epithelium. It is more common in males. The most common site of lesion is the scaphoid fossa and right ear. Though the aetiology remains unclear, it may be associated with mechanical irritation, repeated minor injuries and chronic trauma. If left untreated, it can lead to permanent deformity of the auricle. Pseudocyst of the auricle is a rare and challenging condition. Medical management has unfortunately shown no practical results. Hence, surgical treatment modalities are the best option. The universal aim of treatment is the conservation and restoration of the anatomical structure of the pinna, removal of cystic lesions and prevention of recurrence. Simple aspiration or incision and drainage alone lead to reaccumulation of cystic fluid in most cases. The technique of incision and drainage is the easiest but has a high recurrence rate. Conservative treatment often results in recurrence and unsatisfactory results. Other treatment modalities include incision and drainage with daily irrigation, auricular splinting, aspiration with intralesional steroid injection, aspiration followed by compression dressing, or aspiration with intralesional steroid injection followed by compression dressing and surgical deroofing, surgical deroofing followed by compression with buttons or sheet or sandwich method. Cysts with a diameter less than 1 cm are dealt with through non-surgical treatment modalities, while for cysts having a diameter greater than 1 cm, surgery is considered as first choice therapy. These treatment options have shown promising results. Various modifications in the traditional techniques have reduced the recurrence rate, successfully treated the condition and shown good cosmetic results. This review article aims at providing a holistic collection of various conventional treatment modalities and novel modifications introduced, which can be used in a sequence of definitive treatments of seroma of the auricle.

## Introduction and background

Auricular seroma is a rare, non-inflammatory, asymptomatic, and benign condition of the auricle. The other synonyms of auricular seroma are auricular pseudocysts, cystic chondromalacia, endochondral pseudocyst, intracartilaginous cyst, and benign idiopathic cystic chondromalacia [1,2]. While there is no genetic predisposition, it is more common in Chinese and Caucasian males [2]. The prevalence is more common in males than females and is usually unilateral [3,4]. The hypothesis suggesting hormonal influence affecting the inflammatory process attempts to explain the prevalence in males [5]. In unilateral conditions, the lesions appear more frequently on the right ear than on the left ear [2]. The bilateral presence of seroma is reported in the pediatric group [3]. While seromas can occur anywhere on the auricle, it is more common in the scaphoid fossa [6,7]. Auricular seromas are an intracartilaginous accumulation of sterile, viscous straw-yellow fluid without cells.

The auricle develops from six tubercles around the first and second branchial arches. Defect in the development of these arches leads to the development of residual tissue planes within the cartilage, which may reopen and lead to seroma formation [8]. Intracartilaginous space is formed due to dysplasia of auricular cartilage. Fluid accumulation occurs and results in the formation of pseudocysts [9-11]. Histological examination of the seroma of the auricle suggests the presence of thinned cartilage, granulation tissue and hyalinising degeneration, which lines the cystic space and inflammatory cells. The cystic space is intracartilaginous with no epithelial lining. Analysis of the cyst indicates that the cystic contents are fluid-rich in albumin, cytokine milieu and acid proteoglycans. On analysis, reports indicate elevated interleukin 6 (IL-6) and serum lactic dehydrogenase levels. IL-6 is known to stimulate the proliferation of chondrocytes [3]. On analysis of the autoimmune basis, it was found that the content of immunoglobulin (Ig) G, IgA, IgM and C3 is significantly lower in cystic fluid than in the serum [12]. Elevated levels of serum lactic dehydrogenase support the concept of the development of cysts following repeated minor trauma [13]. Repeated minor trauma may lead to the release of enzymes from degenerated auricular cartilage. Minor trauma or trauma that leads to fragmentation of auricular cartilage can result in the formation of a pseudocyst [14-16]. Medical management with systemic steroids has shown irregular results. The role of systemic steroids is controversial. Patients were treated successfully in a study conducted by Job and Raman [17], but unsuccessfully in a study conducted by Glamb and Kim [18]. The success rate is not consistent. Medical management has unfortunately shown no practical results. It is usually ineffective with a high recurrence rate [19]. The universal aim of treatment is the conservation and restoration of the anatomical structure of pinna, removal of cystic lesions and prevention of recurrence [3,20]. In the absence of treatment, fibrosis and cartilage hardening occur, and it may lead to permanent cauliflower ear deformity, which is challenging to treat [3].

## Review

Surgical deroofing

The method should be performed under local anaesthesia. A helical incision is made based on the position of the seroma. The skin flap is raised until the outermost layer of the seroma is exposed. The cyst is excised along the margin of the pseudocyst, and the fluid is drained. Curettage of the posterior wall of seroma is done to remove granulation tissue and soft tissue debris. To compress the elevated skin flap to its original position, it is sutured with 4-0 black silk sutures. The Vaseline gauze is moulded to a circular shape and sutured with 3-0 horizontal mattress nylon sutures. After one week, removal of the sutures is carried out [21]. According to various reviews, the most preferred and successful treatment option is anterior deroofing of seroma. The method has a 96% success rate [22]. This method requires higher surgical skill sets. A sclerosing agent like minocycline and open deroofing is another recommended method. Cystic content has an elevated level of IL-6. It is advocated that minocycline inhibits the formation of cytokine making it anti-inflammatory sclerosing agent which could provide additional benefit in treatment [23,24].

Surgical deroofing with compression by buttons

Another way to compress the elevated skin flap onto the cartilage is to suture two shirt buttons. Lim modified surgical deroofing by introducing buttoning as a compression method [25]. One button is sutured on the anterior surface and the other on the posterior surface of the pinna with a 2/0 silk suture on a straight needle. Following one week of the procedure, the administration of anti-inflammatory drugs and antibiotics is carried out. One week after the procedure, the buttons and sutures can be removed. This method is known as surgical deroofing, followed by compression of buttons. Studies have shown that this procedure conserves and restores the anatomical structure of pinna with excellent cosmetic results, removes cystic lesions and has almost nil recurrence [26]. Considering this, many other reviews recommended it as a first-line treatment.

Surgical deroofing with compression by PVC-derived sheet

A button cannot be used as a compression material in all cases. Concavities and depression of the auricle make the auricular area irregular. The button bolster may fail to cover the desired area. Due to irregular compression, the chances of recurrence increase. It is also challenging to find a button of the appropriate size [25]. Hence, a substitute for the button was found. The use of plastic sheets as a compression material showed more promising results. The infusion set is readily available, inexpensive, safe, and reliable, and the plastic sheet obtained can be shaped to desired size and shape to provide appropriate compression pressure on both sides of the lesion. The plastic sheet has little impact on the skin and is unlikely to alter the auricular skin's local blood supply. The plastic sheet's transparency makes it easy to see the wound. None of the complications, such as ear fullness, earache or hearing decline are observed on compression with plastic sheet. The plastic sheet material can be obtained from the funnel of the infusion set. It is made up of polyvinyl chloride. It can be cut to the appropriate shape and size. Two sheets are taken. One is sutured to the anterior and the other to the posterior surface of the pinna. No use of an external bandage is made. The wound is allowed to dry as it is kept exposed. After five to six days, the plastic sheet is removed. One week following the operation, the stitches are removed. According to a study, this method has a 98.8% success rate [27].

Surgical deroofing by sandwich method

Another method is the sandwich procedure using a cotton ball and rubber tourniquet sheets. The cotton ball is condensed and moulded to the appropriate size and shape. The cotton ball is submerged in a povidone-iodine solution. The cotton ball is then used to compress the desired area. Two rubber tourniquet sheets are taken. One has to be sutured anteriorly to the cotton ball and the other to the posterior surface of the pinna. It is sutured with two vertical Donati stitches using 2-0 silk sutures in a transfixed manner. This arrangement caters to the need for appropriate and equal compression on the affected area. For the following week after the procedure, analgesics along with antibiotics should be administered. After three days, the cotton ball is removed. One week following the operation, the stitches are removed. According to a study, this method has a 98% success rate. This technique has advantages over other techniques. It is easy to mould the cotton ball to desired size and shape and compress it on irregular concavities and depression of the auricle. This irregularity leads to uniform compression. The cotton ball increases patient compliance by preventing stiffness caused by the sheets. This method's complications could include contact dermatitis, temporary skin staining, and temporary post-operative pain. The patient is prescribed analgesics to decrease the temporary pain after the procedure. A patient who is allergic to rubber or iodine may develop contact dermatitis [28].

Aspiration with intralesional steroid injection

Aspiration with intralesional steroid injection is a simple, short, minimally invasive technique that can be done in the outpatient department (OPD). A 23-gauge needle is used to perform aspiration with a 3 mL syringe. The needle is retained in situ, and the syringe with the aspirated fluid is detached. The needle is kept in place to avoid the second prick and sagging of intracystic space. Another syringe containing 1 mL of 40 mg triamcinolone acetonide solution is attached to the needle, and the solution is injected through the same needle. The steroid accumulates in the subperichondrial space. A spirit swab is pressed at the needle puncture orifice to prevent bleeding and leaking out of medicine. No analgesics and antibiotics are given. Neither any external dressing nor pressure dressing is applied. The doses are to be limited to three to prevent complications due to multiple steroid injections. The procedure can have serious complications like skin pigmentation, perichondrial abscess, atrophy of pinna, cartilage deformity or perichondritis. To prevent perichondritis, oral ciprofloxacin 500 mg is prescribed twice a day for seven days. Following the procedure, if the patient reports of collection of fluid, an additional 40 mg triamcinolone weekly, three doses maximum in total. In some studies, thickening of the pinna was reported, but it was not considered a significant gross cosmetic deformity; hence the result was satisfactory. A progressive increase in the size of swelling at the needle puncture orifice may be reported till the fourth and fifth day. After that, it starts reducing. By the end of the first week, the swelling disappears. Studies promise that it is efficient, cost-effective and a promising treatment modality that avoids recurrence and complications [29,30].

Aspiration and compression dressing using silicone-derived material

This method is simple, noninvasive and inexpensive. The patients undergo aspiration of the pseudo cyst with an 18-gauge needle. A compression patch made up of silicone-derived material is used. This dressing is removed after two weeks. The patient is asked to follow up every month for six months. Studies have shown that this method is highly efficient, with a 92.86% success rate with almost nil recurrence. Recurrence can be due to ineffective pressure. In such cases, the patient retreated with a proper compression dressing [31].

Aspiration followed by intralesional steroid injection and clip compression dressing

A 27-gauge needle is used to perform aspiration with an insulin syringe. The needle is retained in situ, and the syringe with aspirated fluid is detached. Another syringe containing 0.5 mL of 5 mg/mL triamcinolone acetonide solution is attached to the needle, and the solution is injected through the same needle. A gauze dressing is done. Three U-shaped, curved clips retain the dressing. This leads to compression. This procedure is simple, short, and minimally invasive. It can be carried out in office-based clinics as well. It can be done to manage recurrent cases of seroma of the auricle [32].

Incision and drainage with daily irrigation

The method is performed under local anaesthesia. Incision is made on the lower and upper ends of the swelling. The fluid present in the swelling is squeezed out and dissection is done inside by using a hemostat. Drained is passed from the upper incision and let out through the lower incision. The lower and upper ends of the drain catheter are kept free. The upper part of the drain catheter is stitched with one nylon 3-0 stitch to hold the drain catheter in its position. If the seroma is large, lower part of the drain catheter is stitched. The drain catheter has two to three orifices on its body to carry out daily irrigation with Betadine and sodium chloride 0.9%. Vaseline gauze protects the auricle from the edges of the drain catheter. After the removal of the contents of the cyst, mastoid dressing is carried out. The dressing is changed daily. The mastoid dressing is applied every time after the gauze dressing. With a 3mm syringe through the drain, irrigation is done with betadine and sodium chloride 0.9% for 10 days. Daily irrigation averts infections and removes reaccumulating fluid. According to a study, this method has been proven effective without complications. This method has shown satisfactory cosmetic results and nil recurrence during a follow-up period of six months [33].

Auricular splinting

The method is performed under the aural block. An incision is made parallel to the crus of the antihelix. The fluid in the swelling is squeezed out. The blunt end of the freer elevator is used to break septations at the location of the incision. The wound is drained with Betadine wash. Then a corrugated rubber sheet is taken, and it is cut to the appropriate shape and size. This is necessary so that the lesion is covered. The triangular slit is made in an anterior splint to carry out drainage. A curvilinear cut is made in a posterior splint to fit into the post-aural groove. This splint covers the affected pinna anteriorly and posteriorly using 3-0 Ethilon reverse cutting. After the operation, the lesion with a splint is kept exposed. For the following two weeks, oral antibiotics and analgesics are administered. The patient has to follow up for six months. The complications of this method could be discolouration of the pinna, skin thickening, and deformities of the pinna. The discolouration and thickening can be treated with topical emollients, and satisfactory results are obtained in two weeks. Deformity of the auricle, such as skin ridging, resolved in the following two to three weeks. According to the study, this method has a 100% success rate [34].

Cruciate incision

The method is performed under local anaesthesia. Local anaesthesia is administered to block the auriculotemporal nerve and great auricular nerve. Over the most dependent part of swelling, the cruciate incision is made. The flaps are then raised. The accumulated fluid is drained, followed by scraping under the surface of the flap with Rosen’s knife. An appropriate compression dressing is done. Follow-up for three months is recommended. This procedure can have complications like perichondrial reactions with pain and inflammation. To control this complication, analgesics and antibiotics are administered. Other complications include the thickening of pinna at the site of the incision, which resolved over a period of three months. Studies have shown that it has no recurrence, negligible cosmetic deformity and negligible scar marks after three months post-operation [35]. 

Table 1 depicts various treatment modalities for seroma of audible and associated complications.

**Table 1:** Treatment Modalities and Observed Complications

Method	Complications observed
Surgical deroofing	No complications observed
Surgical deroofing with compression by buttons	No complication observed
Surgical deroofing with compression by PVC derived sheet	Sheets may cause stiffness
Surgical deroofing by sandwich method	Contact dermatitis, temporary skin staining, temporary post-operative pain
Incision and drainage with daily irrigation	No complication observed
Aspiration with intraluminal steroid injection	Skin pigmentation, perichondrial abscess, atrophy of pinna, cartilage deformity, perichondritis
Aspiration and compression dressing using silicone derived material	No complications observed
Aspiartion followed by intralesional steroid injection and clip compression dressing	Recurrent cases
Auricular splinting	Discolouration of the pinna, skin thickening, deformity of pinna such as skin ridging
Cruciate incision	Perichondrial reaction with pain and inflammation, thickening of pinna, negligible cosmetic deformity, negligible scar mark

## Conclusions

Pseudocyst of auricle is a rare benign condition. Different treatment modalities have been listed in various reviews. Widely used treatment modalities are surgical deroofing, aspiration with compression dressing, incision and drainage and intralesional steroid injections. Among these, surgical deroofing with compression by sheet has the highest cure rates. Other methods have similar cure rates. Many review articles consider surgical deroofing as a standard treatment modality.
